# Does coevolution promote species richness in parasitic cuckoos?

**DOI:** 10.1098/rspb.2009.1142

**Published:** 2009-08-19

**Authors:** Oliver Krüger, Michael D. Sorenson, Nicholas B. Davies

**Affiliations:** 1 Department of Zoology, University of Cambridge, Downing Street, Cambridge CB2 3EJ, UK; 2 Boston University, Department of Biology, 5 Cummington Street, Boston, MA 02215, USA

**Keywords:** comparative analysis, parasite–host coevolution, speciation, subspecies, virulence

## Abstract

Why some lineages have diversified into larger numbers of species than others is a fundamental but still relatively poorly understood aspect of the evolutionary process. Coevolution has been recognized as a potentially important engine of speciation, but has rarely been tested in a comparative framework. We use a comparative approach based on a complete phylogeny of all living cuckoos to test whether parasite–host coevolution is associated with patterns of cuckoo species richness. There are no clear differences between parental and parasitic cuckoos in the number of species per genus. However, a cladogenesis test shows that brood parasitism is associated with both significantly higher speciation and extinction rates. Furthermore, subspecies diversification rate estimates were over twice as high in parasitic cuckoos as in parental cuckoos. Among parasitic cuckoos, there is marked variation in the severity of the detrimental effects on host fitness; chicks of some cuckoo species are raised alongside the young of the host and others are more virulent, with the cuckoo chick ejecting or killing the eggs/young of the host. We show that cuckoos with a more virulent parasitic strategy have more recognized subspecies. In addition, cuckoo species with more recognized subspecies have more hosts. These results hold after controlling for confounding geographical effects such as range size and isolation in archipelagos. Although the power of our analyses is limited by the fact that brood parasitism evolved independently only three times in cuckoos, our results suggest that coevolutionary arms races with hosts have contributed to higher speciation and extinction rates in parasitic cuckoos.

## 1. Introduction

Explaining the uneven distribution of species richness among taxa is one of the fundamental problems in evolutionary biology (May 1988). Whereas the roles of geographical isolation and ecological specialization in generating reproductive isolation and ultimately speciation are well understood (Darwin 1859; Schluter 2000; Coyne & Orr 2004), the potential of coevolutionary processes to generate biodiversity has so far not been fully explored. Birds are particularly well suited for examination of the role of ecological and behavioural factors in speciation (Price 2008), because of the unparalleled knowledge accumulated on many aspects of their behaviour and distribution. Therefore it is not surprising that they have been influential in shaping the theory of evolution in general (Darwin 1859) and speciation in particular (Price 2008). Understanding the causes and consequences of speciation takes on added importance as current threats to biodiversity call for a better understanding not only of the factors leading to biodiversity loss through extinction, but also its generation through speciation (Coyne & Orr 2004; Janz *et al.* 2006).

For brood parasites and their hosts, coevolutionary processes can exert powerful reciprocal selection, leading to remarkable adaptations and counter-adaptations (Davies & Brooke 1989*a*,*b*; Rothstein 1990; Davies & Welbergen 2009). With increasing fitness costs of parasitism, selection for host defences increases, which in turn may force parasites to specialize and evolve fine-tuned adaptations that overcome a particular host's defences (Davies 2000). Host-specificity in parasites may be achieved either by genetic linkage of traits influencing host use and mate or habitat choice (Hawthorne & Via 2001), or by behavioural imprinting (Payne *et al.* 2000; Sorenson *et al.* 2003). Over time, this process may lead to the formation of host-specific races (Gibbs *et al.* 2000; Starling *et al.* 2006) and eventually new parasite species (Davies 2000; Janz *et al.* 2006). This has been well documented for the host-specific African indigobirds *Vidua* sp., in which speciation is associated with host shifts (Sorenson *et al.* 2003). On the other hand, some hosts may evolve defences that a parasite cannot overcome (Honza *et al.* 2004), leading to the extinction of their associated parasitic species or subspecies (Davies 2000). Therefore coevolutionary arms races might be associated with both elevated speciation and extinction rates.

**Figure 1.**
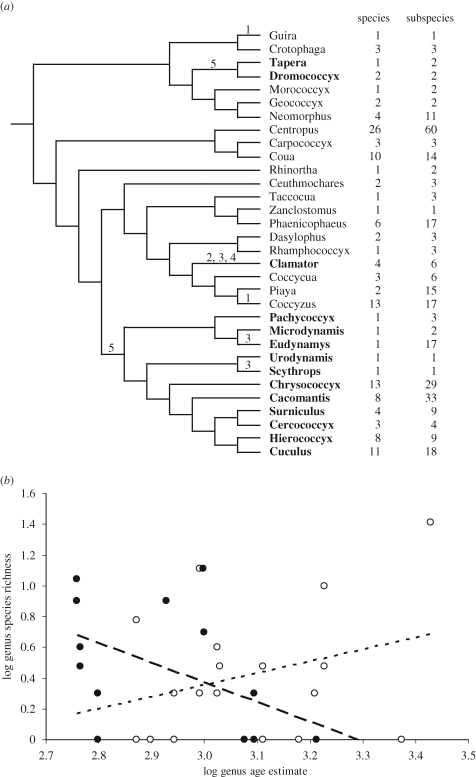
(*a*) Genus-level phylogeny (Sorenson & Payne 2005) of the cuckoo family and (*b*) the relationship between the age of a genus (estimated from the number of nucleotide substitutions per site) and its species richness. Numbers next to the phylogeny (*a*) show the number of species per genus and the total number of subspecies per genus. Parasitic genera are in bold (New World parasitic cuckoos of the genera *Tapera* and *Dromococcyx*, the crested cuckoos of the genus *Clamator* and the subfamily Cuculinae, genera *Pachycoccyx* to *Cuculus*) and numbers above phylogenetic branches show where changes in brood parasitism most parsimoniously arose: 1 = facultative brood parasitism; 2 = obligate brood parasitism, some host chicks can survive; 3 = same as 2 but with egg mimicry; 4 = same as 3 but with ‘mafia’ tactics (Soler *et al.* 1999); 5 = obligate brood parasitism, but with host chick eviction or killing. Dotted line, parental care; dashed line, brood parasitism.

The cuckoos (family Cuculidae) provide a broad perspective, both taxonomically and geographically, to test whether the evolution of host defences might promote specialization and speciation in brood parasites. The family includes 82 species with parental care and 59 species that are obligate brood parasites, distributed across all continents except Antarctica and varying in virulence and also in the variety of hosts that they parasitize (Payne 2005). For example, the olive long-tailed cuckoo *Cercococcyx olivinus* has only one confirmed host, whereas more than 100 are known for the common cuckoo *Cuculus canorus* (Davies 2000; Payne 2005). Obligate brood parasitism has arisen three times independently in cuckoos (Sorenson & Payne 2005), namely in the New World parasitic cuckoos (genera *Tapera* and *Dromococcyx*), in the crested cuckoos (genus *Clamator*) and in the subfamily Cuculinae (genera *Pachycoccyx* to *Cuculus* in figure 1*a*). These independent evolutionary events and the variation in brood parasitism strategy within each lineage provide sufficient variation for comparative analyses (Krüger & Davies 2002; Krüger *et al.* 2007). The parasitic taxa differ greatly in their virulence (figure 1*a*); although the young cuckoo is regularly raised alongside the host chicks in *Clamator* cuckoos, the other two parasitic lineages reduce host breeding success to zero. The New World parasitic cuckoos achieve this by stabbing host chicks to death with sharp hooks on their bills, whereas the parasitic cuckoos within the Cuculinae balance host eggs and chicks on their backs and eject them one by one from the host nest (Davies 2000; Payne 2005). In two instances, Cuculinae genera have evolved less harmful parasitism strategies from an ancestor that was more harmful to hosts, most likely reflecting a constraint on host chick ejection because they parasitize large hosts (Davies 2000). These cases involve some *Eudynamys* cuckoos and *Scythrops*, in which offspring are raised alongside the young of the host.

In this study, we use cuckoos as a model system for examining the role of the coevolutionary process as a promoter of speciation. We hypothesize that powerful selection pressures from hosts have contributed both to the historical diversification of parasitic lineages and to the potential for future speciation as estimated by subspecific diversity. Our analysis tests for the importance of coevolution as a promoter of higher species richness in comparison with other facets of natural and sexual selection. We use comparative analyses, controlling for phylogenetic inertia by calculating independent contrasts (Felsenstein 1985; Purvis & Rambaut 1995), defined by a complete phylogeny of all cuckoos (Sorenson & Payne 2005). The independent evolutionary changes in the brood parasitic behaviour of cuckoos (figure 1*a*) provided us with an opportunity to test whether increased virulence leads to increased levels of species richness and incipient speciation as a product of coevolutionary arms races with hosts.

## 2. Material and methods

### (a) Data collection

We extended the data used in Krüger and Davies (2002, 2004) and Krüger *et al.* (2007), which comprised 12 variables describing cuckoo morphology (body weight, sexual plumage dimorphism, sexual size dimorphism), life history (breeding strategy, egg size, breeding season length) and ecology (diet, breeding habitat, habitat productivity, migration pattern, breeding range size and population abundance), with one variable describing parasite–host association (degree of host diversity) for all 141 recognized cuckoo species (Payne 2005). In addition, we treated one subspecies, *Centropus sinensis andamanensis*, separately, because it is often recognized as a distinct species (Payne 2005), and also separated the two subspecies of *Clamator jacobinus* and *Eudynamys scolopacea* as they have different parasitism strategies (Krüger & Davies 2002). The total number of taxa in our raw database was therefore 144.

In the context of the present study, we ranked the breeding strategy of cuckoos according to the cost imposed on host fitness: 0 = parental care or cooperative breeding (no hosts involved); 1 = facultative brood parasite (host nests used occasionally, infrequent costs to hosts); 2 = obligate brood parasite, host chicks often survive, no egg mimicry (low to moderate costs to hosts, little evidence of evolved host discrimination of eggs); 3 = obligate brood parasite with egg mimicry, host chicks can survive (moderate costs to hosts as evidenced by the evolution of egg discrimination); 4 = obligate brood parasite with egg mimicry, hosts chicks can survive, ‘mafia’ tactics (Soler *et al.* 1999; moderate to high costs to hosts imposed by adult cuckoo destroying host clutches if the host rejects the cuckoo egg); 5 = obligate brood parasite with host chick eviction or killing (high costs to hosts, successfully parasitized nests produce no host young). Use of the original ranking system used in Krüger & Davies (2002) led to qualitatively similar results and identical conclusions. To estimate the degree of host diversity, we used four broad categories for each parasitic cuckoo species: 1 = 1–5 host species parasitized; 2 = 6–10 host species parasitized; 3 = 11–30 host species parasitized; 4 = >30 host species parasitized (see appendix in the electronic supplementary material for data). If species were assigned the same category of host specialization, we used the best estimates of actual number of host species (Payne 2005) to determine the direction of the contrast.

In the few instances where data were missing owing to lack of information, we replaced the missing data with genera-specific means. Excluding cases with missing data from the analyses did not change any of our results and conclusions qualitatively.

### (b) Phylogenetically controlled analyses

We used both genus- and species-level phylogenies based on mitochondrial DNA sequence data (Sorenson & Payne 2005). To test for correlates of realized species richness, we calculated contrasts in MacroCAIC (Agapow & Isaac 2002), a version of CAIC (Purvis & Rambaut 1995) that deals specifically with species richness differences between taxa. As a measure of species richness difference, we used the relative rate difference, which is given by ln(*S_i_* *S_j_*) with *S_i_* and *S_j_* being the species number of taxa *i* and *j*, respectively (Agapow & Isaac 2002). The direction of the contrast was determined by a dummy variable so that the index could be either positive or negative. For analyses involving genera, we calculated genera-means for all independent variables. Because brood parasitism evolved independently only three times in the cuckoo phylogeny, we assumed that its evolution cannot be modelled using Brownian motion, so ancestral trait values were reconstructed using a maximum likelihood approach. This approach reduced the number of non-zero contrasts in breeding strategy significantly, but is a more plausible mode of evolutionary change in this case.

As a more powerful alternative, we used the cladogenesis test developed by Bokma (2003). This approach estimates speciation (*λ*) and extinction (*μ*) rates from the distribution of extant species across genera while taking account of the different ages of the genera. We used branch lengths (in nucleotide substitutions per site) to estimate the relative age of a genus using the complete species-level phylogeny (Sorenson & Payne 2005). The cladogenesis test then proceeded to test whether the estimated speciation and extinction rates differ between non-parasitic and parasitic clades by means of a likelihood ratio test that is *χ*^2^-distributed with two degrees of freedom (Bokma 2003).

To test for correlates of subspecies richness, we used the number of recognized subspecies in Payne (2005) and used the cladogenesis test of Bokma (2003) to see if parasitic cuckoos have higher subspecies speciation and extinction rates than cuckoos with parental care. We then tested for associations with our variables describing parasite–host coevolution by calculating independent contrasts in CAIC. With this, we assumed that the number of cuckoo subspecies is a reasonable proxy for the degree of genetic fragmentation and that its evolution can therefore be modelled following Brownian motion. Subspecies have been morphologically determined, predominantly in the nineteenth century, and hence are independent of information on known hosts, data that typically became available decades after subspecies were described (Payne 2005). The direction of contrasts was determined by a dummy variable and we assumed continuous trait evolution, so ancestral trait values were estimated as weighted means of the sister taxa. Despite subspecies being potentially problematic as an estimate of incipient speciation (Zink 2004), they are useful as an index of diversification and as a surrogate measure of genetic fragmentation (Sol *et al.* 2005; Phillimore *et al.* 2007), which may represent an early stage in allopatric speciation (Phillimore & Owens 2006). Although they have traditionally been defined morphologically based on allopatric phenotypic discontinuities, a recent study found that over 35 per cent of avian subspecies showed considerable phylogenetic differentiation at mitochondrial loci (Phillimore & Owens 2006).

To provide insight into the most likely direction of causality of the relationship between cuckoo subspecies' richness and the diversity of hosts used, we used the reciprocal sister group comparison method developed by Janz *et al.* (2006). Sister clades were chosen on the basis of differences in either host species number or cuckoo subspecies richness. In some cases an increase in cuckoo subspecies number was not accompanied by an increase in the number of hosts used or *vice versa*: if the number of mismatches is not distributed evenly across the two reciprocal trait correlations, this can then be used to test for a significant difference in the distribution of the two reciprocal correlations using a sign test. This test thereby provides an indication of which pathway of causation is more likely (Janz *et al.* 2006).

As some of our variables, such as breeding strategy, represent discrete strategies rather than a continuous spectrum, we categorized contrasts and used analysis of variance (ANOVA) for analysis. We categorized negative contrasts as −1, contrasts with no change in the variable as 0, and positive contrasts as 1. By using only the direction of a contrast and not its magnitude, results are not dependent on a quantitative interpretation of our scoring of breeding strategy and other categorical variables with multiple levels.

Another implicit assumption when calculating independent contrasts is that traits have a heritable component and phylogenetic signal (Blomberg *et al.* 2003). We tested explicitly for phylogenetic signal using nested ANOVA (Harvey & Pagel 1991) to partition total variation into species, genus and subfamily components. A high proportion of the total variation at the species level indicates weak phylogenetic signal, whereas a high proportion of the total variation at higher levels indicates phylogenetic signal and the need to correct for phylogeny.

## 3. Results

### (a) Comparing species richness in parental and parasitic cuckoos

At first sight, there seems to be little evidence for a positive association between the evolution of brood parasitism and species richness, as there are 59 parasitic cuckoo species but 82 species with parental care (figure 1*a*). Comparing the species richness of the New World parasitic cuckoos (*Tapera* and *Dromococccyx*, three species) with their sister clade (*Morococcyx*, *Geococcyx* and *Neomorphus*, seven species) reveals higher species richness in the non-parasitic clade. This is also true for the comparison between the brood-parasitic *Clamator* cuckoos (four species) and their sister clade (*Coccycua*, *Piaya* and *Coccyzus*, 18 species). Only the brood-parasitic subfamily Cuculinae (52 species) has higher species richness than its sister clade (35 species).

Genera with parental care, however, are significantly older than brood-parasitic genera (*F*_1,30_ = 5.389, *p* = 0.027), and so have had more time to speciate. Species richness increases non-significantly with genus age in genera with parental care (*r*_16_ = 0.297, *p* = 0.231), whereas it tends to decrease with genus age in brood-parasitic genera (*r*_12_ = −0.482, *p* = 0.081). Comparing the relationships for parental and parasitic genera reveals a significant difference in slope (figure 1*b*, analysis of covariance, *F*_1,30_ = 4.921, *p* = 0.035). These results suggest large differences in speciation rate between genera with parental care and brood-parasitic genera.

Using the method of Bokma (2003) to compare rates of cladogenesis between non-parasitic and parasitic genera, maximum likelihood estimates of speciation rate (*λ*) were more than twice as high for parasitic genera (0.4003) as for genera with parental care (0.1523). However, most likely estimates for extinction rates (*μ*) were four times higher for parasitic genera (0.3845) than for genera with parental care (0.0875). The difference in the net rate of cladogenesis is highly significant (likelihood ratio test = 6.381, d.f. = 2, *p* = 0.002). Therefore, although brood-parasitic genera have higher rates of speciation, their higher rates of extinction lead to a higher net speciation rate (*λ*–*μ*) in genera with parental care.

**Figure 2.**
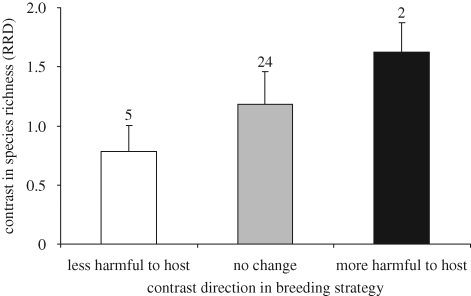
The contrasts in species richness in relation to changes in breeding strategy contrasts across all cuckoo genera. The species richness index (RRD = relative rate difference) is calculated as ln(*S_i_*/*S_j_*), where *S_i_* and *S_j_* are the species number of the two taxa *i* and *j* (Agapow & Isaac 2002). The numbers above the error bars (s.e.) provide the sample sizes.

Reconstructing ancestral states by maximum likelihood, evolutionary changes in breeding strategies towards those more costly to host fitness were associated with increased cuckoo species richness compared with changes towards less costly brood parasitism or contrasts with no change in breeding strategy (figure 2), but with so few evolutionary transitions, differences were far from significant (figure 2, ANOVA, *F*_2,28_ = 0.362, *p* = 0.699).

We examined 11 other variables covering cuckoo morphology (body weight, plumage dimorphism, size dimorphism), life history (egg size, breeding season length) and ecology (diet, habitat, habitat productivity, migration pattern, geographic range size and abundance) as potential correlates of realized differences in species richness levels. However, none showed a significant association with the species richness index across cuckoo genera (*p*-values between 0.166 and 0.897; see apendix in the electronic supplementary material for details). If past speciation events led to fragmentation of ancestral geographic ranges, then we predict that mean geographic range size per genus should correlate negatively with cuckoo species richness. Geographic range size had a strong phylogenetic signal, with only 20.8 per cent of the total variation at the species level, but 33.7 per cent at the genus level and 45.5 per cent at the subfamily level; therefore calculating independent contrasts in geographic range size is warranted. However, there was no relationship between the species richness index and contrasts in mean geographic range size per genus (*r*_27_ = −0.098, *p* = 0.854).

### (b) Correlates of subspecies richness in cuckoos

If subspecies formation is simply a function of time, we would expect a strong positive correlation between subspecies richness and a species' age. Using terminal branch lengths (in nucleotide substitutions per site) to estimate a species' relative age in the complete species-level phylogeny (Sorenson & Payne 2005), we found that subspecies richness was not related to species age (*r*_142_ = 0.087, *p* = 0.302); therefore, other factors must influence subspecies richness in cuckoos. Across cuckoo species, subspecies diversification rate (estimated as log(subspecies number)/species age) was about eight times higher in parasitic cuckoos (0.0047 ± 0.0025) than in cuckoos with parental care (0.0006 ± 0.0001). Interestingly, there was no significant positive correlation between cuckoo subspecies richness and geographic range size for parasitic species (*r*_58_ = 0.094, *p* = 0.476), although there was a significant positive correlation for species with parental care (*r*_82_ = 0.308, *p* = 0.004), emphasizing again that traditional factors such as evolutionary age or geographic range size are poor predictors of subspecies richness in parasitic cuckoos.

Using Bokma (2003) to compare rates of cladogenesis between non-parasitic and parasitic species revealed that the most likely estimates for subspecies speciation rate (*λ*) were more than twice as high for parasitic species (0.8569) than for species with parental care (0.3615). However, most likely estimates for extinction rates (*μ*) were also more than twice as high for parasitic species (0.8426) than for species with parental care (0.3458). These differences are highly significant (likelihood ratio test = 28.037, d.f. = 2, *p* < 0.001). Therefore, brood-parasitic species seem to have higher rates of both formation and extinction of subspecies compared with species with parental care, and estimated rates of net subspeciation (*λ*–*μ*) were similar (brood-parasitic cuckoos = 0.0143 versus cuckoos with parental care = 0.0157).

We then tested whether cuckoo subspecies richness shows a phylogenetic signal. In other words, do species within higher taxonomic units share similar levels of subspecific diversity? In two previous avian studies (Sol *et al.* 2005; Phillimore *et al.* 2007), closely related species differed markedly in subspecies number (75–95% of total variation in subspecies number), whereas differences between genera and subfamily were small (5–25% of total variation). In cuckoos, however, closely related genera differed strikingly in subspecies richness (54.8% of total variation in subspecies richness is at the genus level, with only 38.5% at the species level). Therefore, we needed to control for phylogeny when examining correlates of subspecies richness.

**Figure 3.**
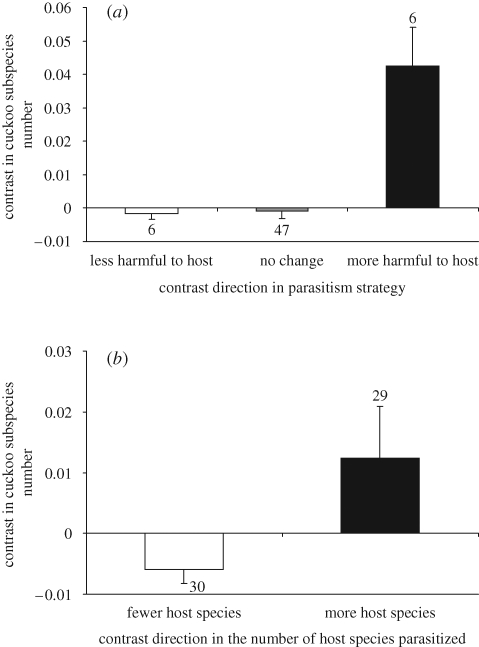
(*a*) The contrast categories in parasitism strategy and (*b*) contrast categories in the number of host species against contrasts in subspecies number across brood-parasitic cuckoo species. For both parts the numbers above error bars (s.e.) provide the sample sizes.

Crucially, contrasts towards a more virulent parasitism strategy were associated with higher contrasts in subspecies number, and contrasts towards a less virulent parasitism strategy, or contrasts with no change in virulence level, were associated with lower contrasts in the number of subspecies (figure 3*a*, ANOVA, *F*_2,56_ = 4.599, *p* = 0.014). Therefore, changes in parasitism strategy towards higher costs to host fitness were associated with increased cuckoo subspecies richness.

### (c) Cuckoo subspecies richness and host diversity

**Figure 4.**
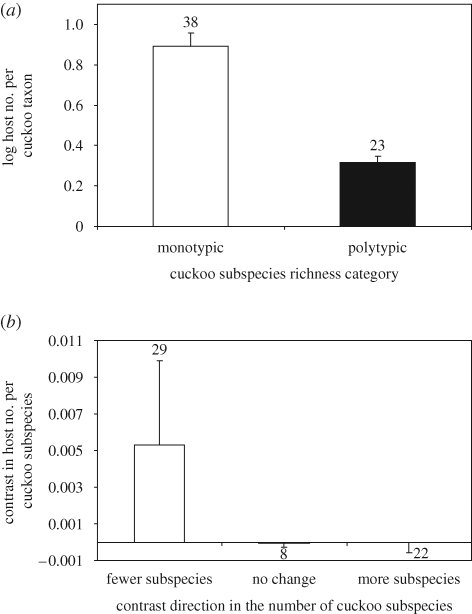
(*a*) Cross-species comparison between monotypic (one subspecies) and polytypic (two or more subspecies) brood-parasitic cuckoo species with regard to the number of hosts per subspecies (data for the number of hosts are log-transformed). Error bars show means plus s.e. (*b*) Contrast categories in cuckoo subspecies number against contrasts in the number of host species per cuckoo subspecies. Bars show means plus s.e.

Across brood-parasitic cuckoo species, monotypic species use, on average, eight host species, whereas polytypic cuckoo species (those with two or more subspecies) use, on average, two host species per cuckoo subspecies (figure 4*a*). This difference is highly significant (*F*_1,59_ = 42.880, *p* < 0.001), suggesting that cuckoo subspecies tend to use fewer host species each compared with monotypic cuckoo species. Although this difference is also apparent using contrasts in the number of host species per cuckoo subspecies (figure 4*b*), the difference is not statistically significant (*F*_2,56_ = 0.681, *p* = 0.510).

If the coevolutionary process is causally associated with both cuckoo subspeciation and host diversity, we would expect cuckoo species that use many hosts to have evolved more subspecies. To test whether the number of host species is related to subspecies richness, we used the entire cuckoo phylogeny (Sorenson & Payne 2005). Species-level variation in host numbers accounted for only 33.3 per cent of the total variance, whereas 62.8 per cent occurred at the genus level and 3.9 per cent at the subfamily level. As closely related genera differed markedly in the number of host species used, we needed to include phylogeny in the comparative analysis. The estimated age of a cuckoo species was not a predictor of the diversity of hosts used (*r*_52_ = −0.125, *p* = 0.368). Changes towards more host species were associated with positive contrasts in cuckoo subspecies richness and changes towards fewer host species were associated with negative contrasts in cuckoo subspecies richness (figure 3*b*, ANOVA, *F*_1,57_ = 4.151, *p* = 0.046).

Clearly, cuckoo species with larger geographic ranges might have both a greater number of subspecies and a greater number of host species, due to parallel effects of isolation on both measures. Indeed, contrasts in geographic range size were positively associated with contrasts in both recognized subspecies richness (*r*_57_ = 0.928, *p* < 0.001) and host number (*r*_57_ = 0.973, *p* < 0.001). In addition, more virulent parasites have larger geographic ranges, as contrasts in parasitism strategy were positively correlated with contrasts in range size (*r*_57_ = 0.990, *p* < 0.001). Therefore, not only parasitism strategy and host specialization but also geographic range size may influence current species richness and the potential for future diversification. To test whether geographic range size could be a confounding variable, we used an information-theoretic approach. All five best models explaining cuckoo subspecies richness contrasts included host diversity (table 1), and the minimum adequate model did not contain geographic range size and had by far the highest model weight. We concluded that host specialization was associated with subspecies richness, independent of geographic range size effects. In addition, the second-best model contained parasitism strategy, so there is evidence that cuckoos imposing greater costs on hosts have higher subspecies richness, independent of geographic range size.

**Table 1.** Multivariate regression models for contrasts in parasitic cuckoo subspecies. Model selection was based on the small sample version of the Akaike information criterion (AICc). The direction of the relationship between independent variables and contrasts in cuckoo subspecies number is given in brackets. All models are highly significant (*p* < 0.001).

model	AICc	ΔAICc	weight	*R*^2^
host diversity (+), altitude (+)	−524.8	0.00	0.177	0.953
host diversity (+), breeding strategy (+), altitude (+)	−523.5	1.35	0.090	0.954
host diversity (+), egg size (−), altitude ( +)	−523.3	1.50	0.084	0.954
host diversity (+), range size (+), altitude (+)	−523.1	1.73	0.075	0.953
host diversity (+), size-dimorphism (−), altitude (+)	−523.1	1.75	0.074	0.953

Island archipelagos are another cause of isolation (Coyne & Orr 2004; Price 2008) and therefore could independently influence both cuckoo and host species richness. Even when all parasitic cuckoo species living on islands or in archipelagos were excluded from the analysis (see Methods and the appendix in the electronic supplementary material), the correlation between contrasts in the number of host species and contrasts in cuckoo subspecies richness remained highly significant (*r*_11_ = 0.999, *p* < 0.001). Therefore, the correlation between more cuckoo subspecies and the number of hosts used was not due solely to effects of increased isolation through increased geographic range size or archipelagos.

### (d) Pathway of causation

Do cuckoo species with more subspecies have a greater opportunity to parasitize novel hosts or does host specialization result in the evolution of more cuckoo subspecies? We used reciprocal sister group comparisons to differentiate between these two hypotheses.

We found 19 valid sister comparisons across the species phylogeny where there are differences in the number of hosts used by parasitic cuckoos. Of these, 12 showed a corresponding change in cuckoo subspecies number (sign test, *p* = 0.263). Using the reciprocal method, there were 14 sister comparisons that differed in cuckoo subspecies number and, among these, 12 showed a corresponding change in the number of hosts used (sign test, *p* = 0.039). Therefore, the association was significant only when using the number of cuckoo subspecies as the basis. The different outcome of the two reciprocal methods of sister clade comparisons suggests that the data are more consistent with the hypothesis that cuckoo subspecies number influences the number of hosts used rather than *vice versa*.

## 4. Discussion

Our results indicate that the evolution of brood parasitism in cuckoos resulted in increased rates of both speciation and extinction. Therefore, coevolutionary arms races might indeed influence the rate of cladogenesis in parasitic cuckoo lineages, but do not necessarily lead to a higher net speciation rate because of parallel increases in extinction rate. Whereas the population dynamics of generalist parasites should be buffered by their use of multiple hosts, the conditions for stable coexistence of a specialist parasite and its host are more restrictive (May & Robinson 1985). Thus, host specialization may promote cladogenesis by producing adaptive differentiation among parasite lineages, but at the same time increase the likelihood of extinction by linking the fate of specialist parasites to single hosts.

Cuckoo species with a larger diversity of hosts may be in the process of further evolutionary diversification, as is made evident by a larger number of described subspecies. Fidelity of female genetic lineages (gentes) to specific hosts has already been documented or implicated in two cuckoo species (Gibbs *et al.* 2000; Starling *et al.* 2006) and is attributed to host–parasite coevolution. Crucially, the relationship between cuckoo subspecies number and host diversity remained significant after controlling for the effect of geographic range size.

Because the evolution of brood parasitism was associated with increased speciation rate but also extinction rate, brood-parasitic genera are not more speciose than genera with parental care. Nevertheless, it seems that coevolutionary arms races can lead to rapid speciation, specialization and ultimately extinction of taxa (Davies 2000). In parasitic finches (genus *Vidua*), colonization of new hosts has resulted in recent and rapid speciation (Sorenson *et al.* 2003), yet the origin of parasitism in this clade is ancient, suggesting the possibility of repeated cycles of cladogenesis and extinction (Sorenson & Payne 2001). Similarly, Wilson (1971), in observing that the most specialized parasitic ants are rare, suggested that ‘they give the impression, quite possibly false, of having no more than a toehold on their host population and of existing close to the edge of extinction’.

Some non-parasitic cuckoo genera such as the coucals *Centropus* are species rich, probably due to colonization of many different islands followed by speciation, so macroecological factors are also clearly important. Another potential macroecological factor in brood-parasitic cuckoos is latitude; species at higher latitudes use more host species (Yom-Tov & Geffen 2005). It has been suggested that this is a consequence of less suitable food for cuckoos at higher latitudes, resulting in fewer cuckoo species, less competition for hosts and therefore niche expansion (Yom-Tov & Geffen 2005). Alternatively, there may have been less time for cuckoo speciation if cuckoos invaded higher latitudes more recently (Rothstein *et al.* 2002). However, we found that the age of a cuckoo species is a poor predictor of cuckoo subspecies richness. Competition for hosts might also have influenced cuckoo species richness (Brooker & Brooker 1990); regions with more suitable host species might allow more cuckoos to coexist.

The association between the breadth of host usage and cuckoo subspecies richness might also be explained by shared ecology rather than coevolution; parasitic cuckoos and their hosts, however, are only linked by reproduction and often occupy very different ecological niches with respect to other traits such as feeding ecology (Davies 2000; Payne 2005). Nevertheless, we found that the most likely causal pathway was that a higher number of cuckoo subspecies leads to a higher number of host species being parasitized, not *vice versa*. Under a coevolutionary arms race scenario, we would expect the opposite causal relationship, so it appears that shared ecology may play a significant role in cuckoo subspecies diversification. A crucial comparative test of whether coevolutionary arms races promote subspeciation would be to test for a phylogenetically controlled correlation between the number of cuckoo gentes and the number of hosts used, but such data are still very scarce as the necessary population-level analyses have not been completed. Although it is well known that many cuckoo species have different egg morphs, to date, genetic host races (gentes) have been documented or implicated for only two cuckoo species (Gibbs *et al.* 2000; Starling *et al.* 2006).

The search for correlates of species richness in birds generally has been plagued by low explanatory power (Phillimore *et al.* 2006). For cuckoos also, we found no additional, significant correlates of species richness across genera, despite testing 12 variables covering coevolution, morphology, ecology and indices of sexual selection. Consistent with another recent study testing for an effect of sexual selection on species richness in birds (Phillimore *et al.* 2006), neither size dimorphism or plumage dimorphism were significant predictors of species richness in cuckoos. We also found no relationship between the age of a cuckoo species and either cuckoo subspecies richness or host diversity. In contrast, the importance of coevolution for shaping cuckoo life histories has been documented for other traits traditionally viewed as prime candidates for sexual selection (Krüger *et al.* 2007).

Our results partially support the hypothesis that coevolution could help to account for both current species richness and the potential for future diversification in brood-parasitic cuckoos. Association with different hosts could, under the right conditions, provide an easy route towards either allopatric or sympatric speciation, as has been shown in the brood parasitic indigobirds (Sorenson *et al.* 2003), in which host songs play an important role in the social behaviour of the parasites (Payne *et al.* 2000). In parasitic cuckoos, mechanistic explanations may include host or habitat imprinting as promoters of reproductive isolation of sympatric individuals associated with different hosts (Payne 2005).

The explanatory power of coevolutionary arms races as selective forces shaping life history evolution in brood parasites and hosts is well recognized (Davies & Brooke 1989*a*,*b*; Rothstein 1990; Krüger 2007; Boerner & Krüger 2008). Our results support the idea that these arms races might also contribute to differences in rates of cladogenesis and extinction between lineages. Remarkable parallels are observed in arms races between phytophagous insects and their host plants, where inclusion of new host plants is associated with higher levels of species richness (Janz *et al.* 2006). Our results are less clear cut, as the most likely causal pathway was that a larger number of cuckoo subspecies leads to a larger number of host species, yet they support the idea that parasitism is a selective force that might promote the generation of biodiversity.
